# High-Fat Diet as a Risk Factor for Breast Cancer: A Meta-Analysis

**DOI:** 10.7759/cureus.32309

**Published:** 2022-12-08

**Authors:** Trinitas Oserefuamen Uhomoibhi, Tobechukwu J Okobi, Okelue E Okobi, Jovita O Koko, Osezimen Uhomoibhi, Ogie E Igbinosun, Uyiosa D Ehibor, Maureen G Boms, Rafiat A Abdulgaffar, Bolaji L Hammed, Chinenye Ibeanu, Esther O Segun, Adeyemi A Adeosun, Endurance O Evbayekha, Kesena B Alex

**Affiliations:** 1 Lombardi Comprehensive Cancer Center, Georgetown University/University of the District of Columbia, Washington D.C, USA; 2 Internal Medicine, BronxCare Health System, New York, USA; 3 Family Medicine, Arizona State University, Tempe, USA; 4 Family Medicine, Lakeside Medical Center, Belle Glade, USA; 5 Family and Community Medicine, University of Jos, Jos, NGA; 6 Internal Medicine, Georgetown University, Bronx, USA; 7 Haemato-Oncology, University of Benin Teaching Hospital, Benin City, NGA; 8 Family Medicine, University of Benin Teaching Hospital, Benin City, NGA; 9 Clinical Research, University of Alabama at Birmingham, Birmingham, USA; 10 Communicable Disease Control, Alberta Health Services, Calgary, CAN; 11 Family Medicine, Plato Hospital, Lagos, NGA; 12 Pediatrics, V.N. Karazin Kharkiv National University, Kharkiv, UKR; 13 School of Biological Sciences & Applied Chemistry, Seneca College, Toronto, CAN; 14 Molecular Pharmacology and Experimental Therapeutics, Mayo Clinic, Rochester, USA; 15 Internal Medicine, St. Luke's Hospital, Chesterfield, USA; 16 Faculty of Medicine, University of London, London, GBR

**Keywords:** risk factors for breast cancer, causes of breast cancer, mortality of breast cancer, breast cancer biology, metastatic breast disease, cancer, obesity and breast cancer, fat diet, high-fat diet, breast cancer

## Abstract

High-fat diets have been identified as a major cause of obesity and a potential risk factor for breast cancer. Fat tissue, also known as adipose tissue, produces an excess of estrogen, which has been linked to an increased risk of breast cancer. Determining the impact of HFDs in the development and progression of breast cancer is essential, as it will enable us to identify the role of dietary modification in preventing and managing the disease.

The impact of a high-fat diet (HFD) on the development of breast cancer in humans has yet to be fully explained, as very few human studies are available to effectively analyze the effect fatty food has on breast cancer development. This meta-analysis, therefore, seeks to determine the strength of association, if any, between HFD and an increased risk of breast cancer development. This research will help inform good eating habits, potentially reducing the disease's incidence and outcome.

This meta-analysis examined eight (8) papers from various nations examining the effect of a high-fat diet as a risk factor for breast cancer development between 2010 and 2020. The study employed the multivariable-adjusted hazard ratio (H.R.), odds ratio (OR), or relative risk (R.R.) from the studies. Breast cancer cases were histologically and radiologically confirmed in the studies evaluated, and validated food frequency questionnaires were used to assess their dietary patterns. This metanalysis study found a substantial link between a high-fat diet and an increased risk of breast cancer, with statistically significant results (I^2^ = 93.38%, p0.05). Changes in dietary fat consumption may thus help mitigate some of the unfavorable consequences of breast cancer and survival. Even if further research is needed to support this assertion, the findings are compelling enough to advocate for low-fat, healthy diets to avoid breast cancer.

## Introduction and background

Breast cancer is the second most common cancer in women worldwide, next to skin cancers, with over 2 million cases observed in 2018 [1]. It is also the second leading cause of cancer deaths among women, next to lung cancer. Each year in the United States, over 250,000 breast cancer cases are diagnosed in women making up about 15% of all newly diagnosed cancer cases and about 2,300 in men. Over 40,000 women in the U.S. die yearly from breast cancer, making up about 7% of all cancer deaths [1-4].

Several factors have been identified to increase the risk of breast cancer development, including genetics, family and personal histories, age, sex, race, lifestyle, and dietary factors. Most breast cancers are found in women aged 50 and above [1-6]. Of the dietary factors, a high-fat diet and, consequently, overweight/obesity have been strongly associated with the disease, although this link is yet to be completely understood. A few studies have demonstrated the association of dietary content as a contributing factor to disease development, including Ziegler et al. [3]. Other studies focusing on migrant populations have shown that women migrating from countries with low breast cancer incidence (Asian, Latin American) to countries with high breast cancer incidence (the United States and other Western countries) have developed breast cancer at a rate similar to those in the new region [3]. This has been shown to be partly due to lifestyle and dietary patterns changes [3-8]. Most Western diets are composed of fat-rich foods. Murtaugh et al., in their study of including women of different ethnicities and their risk of breast cancer in relation to their dietary patterns, found that women consuming a western diet had a higher risk of B.C. [5].

High dietary fat, which is a major cause of obesity, has been proposed as a risk factor for breast cancer [2-8]. Obesity in itself has been documented as a major driver of breast carcinogenesis [2-9]. The association between breast cancer and obesity is explained through numerous complicated signaling pathways that alter cell growth and angiogenesis [6-14]. This complex interaction and the role a high-fat diet (HFD) has on developing breast cancer in humans is yet to be fully understood, partly due to the limited number of human studies [1-4,7-13]. Identifying the strength of the association between HFD and its impact on breast cancer development is crucial, as it could inform dietary and lifestyle changes that will potentially reduce breast cancer incidence and outcome.

Methodology

A PubMed search for the terms "risk factors for breast cancer" and "high-fat diet" produced 49 results. "High-fat diet," "diet," "risk factors," and "breast neoplasm" or "breast cancer" were the MeSH search phrases. After filters were applied, the study's most relevant articles were included based on our eligibility criteria.

Eligibility criteria 

Randomized clinical controlled trials, cohort studies, and case-control studies between 2010 and 2020, which focused on high-fat diet and breast cancer risk were reviewed. Systematic review articles and meta-analyses were excluded. Articles, abstracts, and titles irrelevant to this paper's aim were excluded. Of the 49 articles identified, eight were included in this meta-analysis based on the exclusion and inclusion criteria as listed in Table 1 below.

**Table 1:** Summary of inclusion and exclusion criteria

Inclusion criteria	Exclusion criteria
1) Literature relevant to the role of a high-fat diet as a risk factor for breast cancer.	1) The studies that did not discuss a high-fat diet as a risk factor for breast cancer were excluded because the study's objective was focused on the interrelationships between a high-fat diet and breast cancer.
2) The studies must be original case-control studies, cohort studies, or randomized clinical controlled trials on the interplay of high-fat diet and breast cancer.	2) Opinion pieces, review articles, non-scholarly articles, secondary studies, scoping reviews, and research approaches other than primary studies were excluded.
3) The selected studies may be heterogenous but must have measurable endpoints with calculatable risks like hazard ratio (HR), odd ratio (OR), or relative risk (RR).	3) Studies without calculatable risks - odd ratio (OR), hazard ratio (HR), or relative risk (RR) were excluded.
4) Human studies.	4) Animal studies.
5) The studies must be published in a peer-reviewed journal to maintain the validity and reliability of the studies.	5) The studies that were published in non-peer-reviewed journals, and dissertations were excluded.
6) The studies must be originally published in English for readability by the reviewers.	6) The studies originally published in a language other than English were discarded.
7) Works of literature published between (2010–2020).	

**Figure 1:**
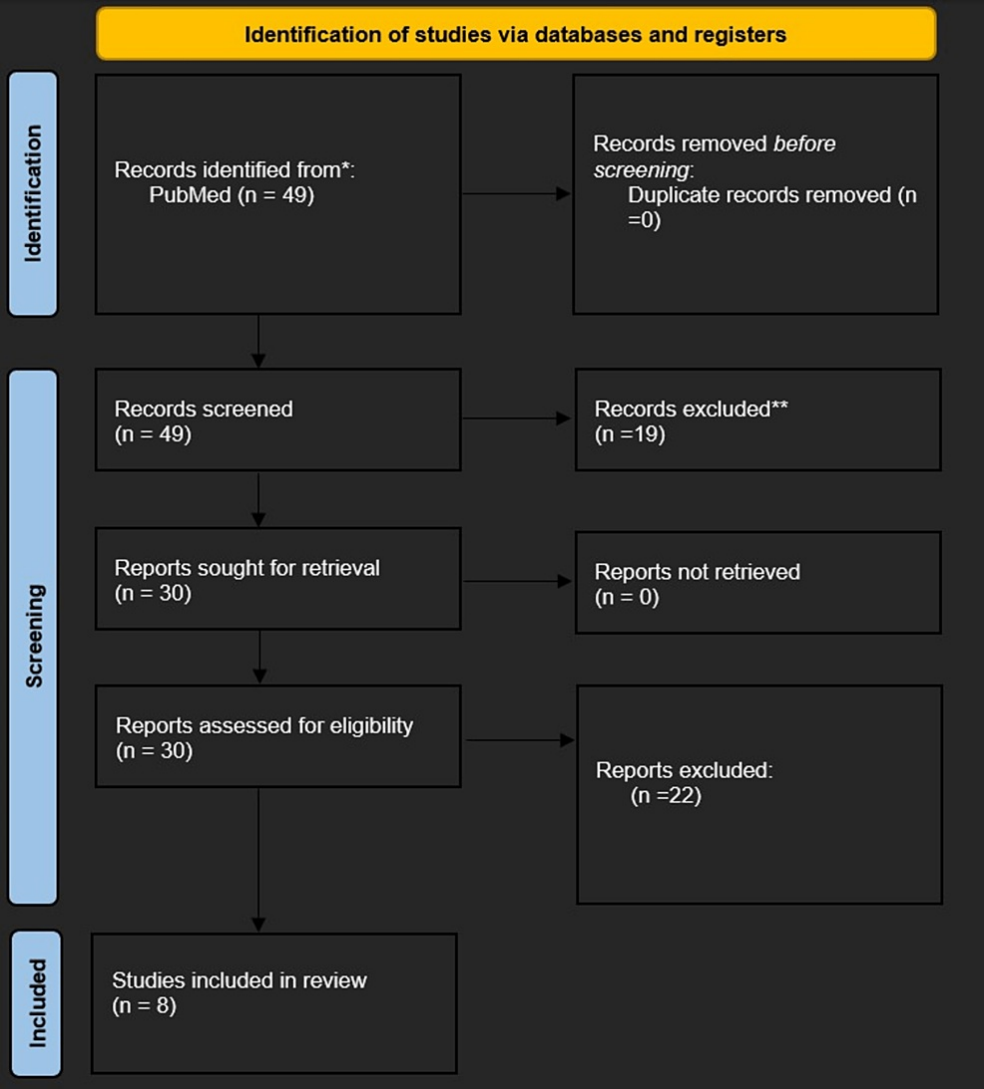
PRISMA flow for inclusion and exclusion criteria PRISMA: Preferred Reporting Items for Systematic Reviews and Meta-Analyses

Figure 1 shows the PRISMA flow diagram.

Data analysis method

The multivariable-adjusted hazard ratio (H.R.), odds ratio (O.R.), or relative risk (R.R.) provided in various studies was used in the data analysis. The specific software used to create the forest plot was STATA 16 (StataCorp, College Station, Texas, USA). Forest plots were created for all studies reviewed. Sub-group analysis was also performed comparing the cohort and case-control studies.

Result

Eight (8) studies from different countries published between 2010 and 2020 were reviewed for this meta-analysis (see Table 2). Of these studies, three were cohort studies (sample sizes - of 201 to 91,779 people) and five were case-control studies (sample sizes of 172 and 9031 people). In these studies, breast cancer cases were diagnosed both radiologically and histologically. Also, validated self-administered questionnaires were used to assess how much of a high-fat diet they consumed compared to other diets. The O.R., H.R., and R.R. from these studies were used in the statistical analysis. Table 2 shows the characteristics of the studies used in this meta-analysis.

**Table 2:** Table of included studies showing the characteristics of the studies used in this meta-analysis

Author	Year of publication	Population of cancer patients	Country	Type of Study	Method of breast cancer assessment	Method of health and dietary assessment	“OR” or “RR” (99% confidence interval)
Fung et al. [8]	2011	3,314	USA	Case-control	Histology	Questionnaire and interview	0.8 (0.64, 1.01)
Kruk, and Marchlewicz [9]	2010	858	Poland	Case-control	Histology	Interview	1.66 (1.0 7, 3.59)
Jones et al. [10]	2014	172	USA	Case-control	MRI	Questionnaire and interview	0.40 (0.3 3, 1.03)
Berkey et al. [11]	2019	9031	USA	Cohort	Histology	Questionnaire	2.27 (1.9 8, 3.87)
Dydjow-Bendek, and Zagoźdźon [12]	2020	201	Poland	Cohort	Histology	Questionnaire and interview	0.40 (0.1 9, 0.85)
Khankari et al. [13]	2016	1506	USA	Case-control	Histology	Questionnaire	0.41 (0.0 6, 0.76)
Link et al. [14]	2013	91,779	USA	Cohort	Histology	Questionnaire	1.29 (1.1 2, 1.49)
Turati et al. [15]	2010	3034	Switzerland and Italy	Case-control	Histology	Questionnaire and interview	0.86 (0.7 6, 0.98)

## Review

Metanalysis

**Figure 2:**
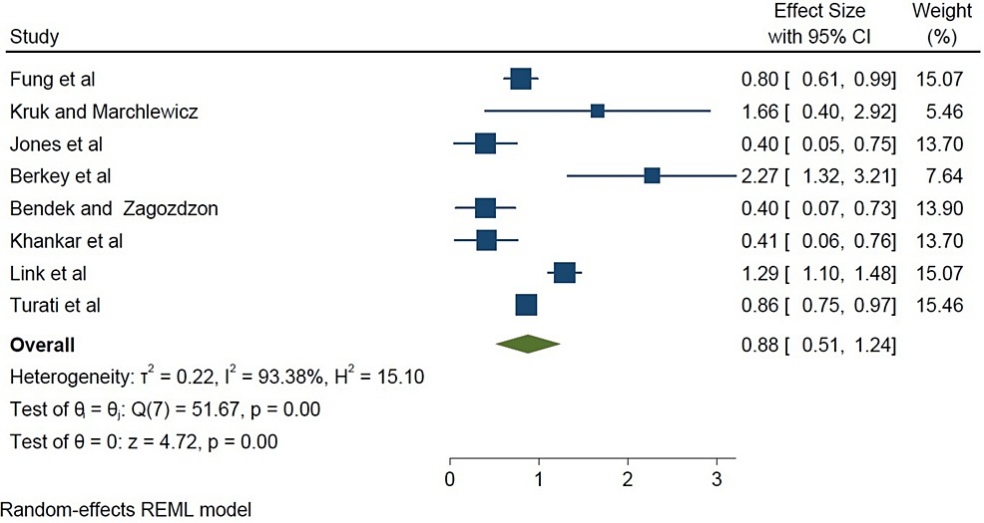
A forest plot of all studies weighted in square sizes demonstrating a strong association between a high-fat diet and the incidence of breast cancer Fung et al. [8], Kruk and Marchlewicz [9], Jones et al. [10], Berkey et al. [11], Dydjow-Bendek and Zagoźdźon [12], Khankari et al. [13], Link et al. [14], Turati et al. [15] REML: restricted maximum likelihood

In this study, we made our plots using the 95% confidence intervals (CI) and multivariate-adjusted OR derived from the individual papers. The square sizes were proportional to each study’s sample size, as shown in Figure 2.

**Figure 3:**
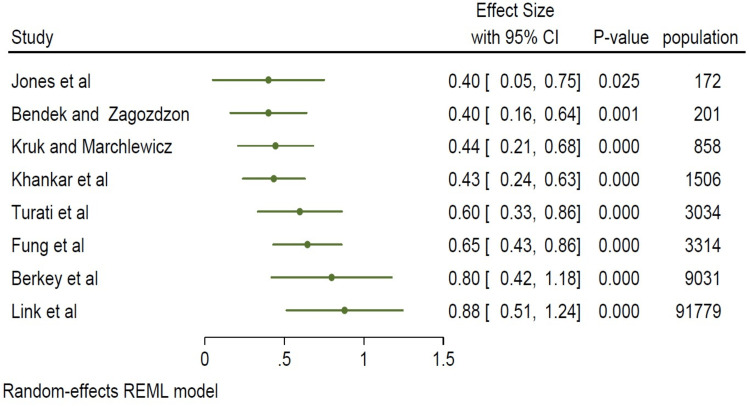
A forest plot of all studies demonstrating a strong association between a high-fat diet and the incidence of breast cancer Fung et al. [8], Kruk and Marchlewicz [9], Jones et al. [10], Berkey et al. [11], Dydjow-Bendek and Zagoźdźon [12], Khankari et al. [13], Link et al. [14], Turati et al. [15] REML: restricted maximum likelihood

In the studies analyzed, there was a statistically significant association between a high-fat diet and breast cancer incidence (I^2^=93.38%, p<0.05), as shown in Figure 3.

**Figure 4:**
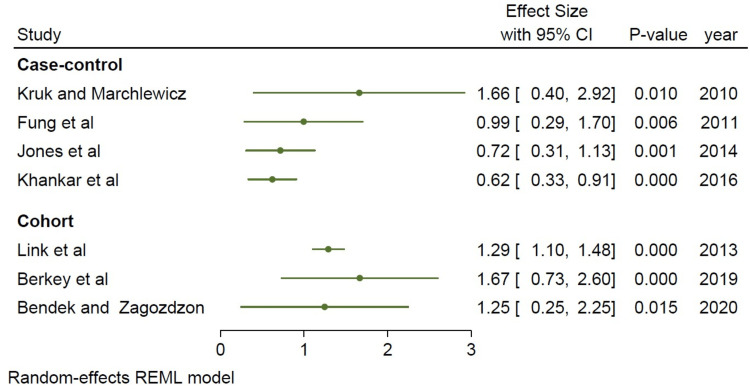
Forest plot of the case-control versus cohort studies Fung et al. [8], Kruk and Marchlewicz [9], Jones et al. [10], Berkey et al. [11], Dydjow-Bendek and Zagoźdźon [12], Khankari et al. [13], Link et al. [14], Turati et al. [15] REML: restricted maximum likelihood

**Figure 5:**
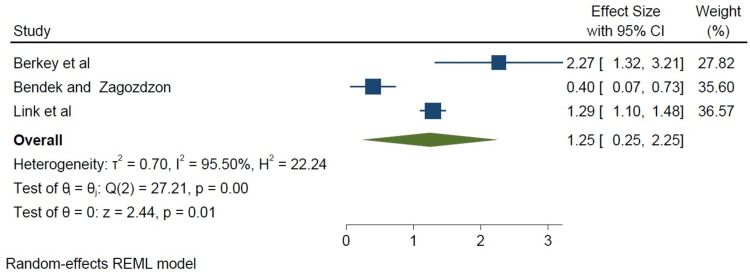
Forest plot of the cohort studies (square weighted) Berkey et al. [11], Dydjow-Bendek and Zagoźdźon [12], Link et al. [14] REML: restricted maximum likelihood

**Figure 6:**
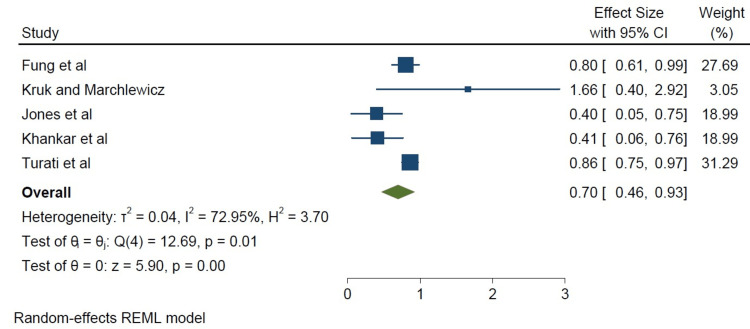
Comparing case-control studies to cohort studies using a forest plot, the cohort studies showed a stronger correlation Fung et al. [8], Kruk and Marchlewicz [9], Jones et al. [10], Khankari et al. [13], Turati et al. [15] REML: restricted maximum likelihood

We did a subset analysis comparing cohort studies and case-control studies based on study type. The cohort studies appeared to show a stronger association between a high-fat diet and breast cancer incidence when compared to the case-control studies, as shown in Figure 4. However, the limited number of studies available for subgroup analysis (five case-control studies, three cohort studies) may be a potential limitation of this study (Figures 4-6). Below are forest plots for the two subgroups.

Although all the studies showed a significant association, a stronger association was found in the cohort studies (I^2^=95.50%, p=0.0001 in Figure 5) compared with the case-control studies (I^2^=72.95%, p=0.01), as seen in Figure 6.

Discussion 

This meta-analysis utilized scientifically validated methods to determine whether there is a strong association between a high-fat diet and breast cancer incidence across the existing literature. The studies reviewed demonstrated a statistically significant association, with some of the studies showing a stronger association than others. Our study agrees with several other well-powered prospective studies and metanalysis supporting the association between breast cancer incidence and a high-fat diet [1-8,16-21].

Numerous cancer causes have been linked to a high-fat diet. High-fat diets include lipids that may be separated into plant vs. animal fat-based fat, then further subdivided into polar and nonpolar molecules such as fatty acids, triglycerides (TGs), monoglycerides, diglycerides, sterols, and phospholipids. The fatty acid component based on the type of carbon bonding (saturated vs. unsaturated), number of double bonds (monounsaturated versus polyunsaturated), location of the double bond (trans versus cis), and chain length (short, medium, or long) [1-8,16-21]. Several studies have shown the role of fatty acids (FAs) in cell proliferation-related signaling pathways while demonstrating the fatty acid-carcinogenic relationship. For instance, overall caloric intake has a larger impact on the development of obesity, which is linked to redox and hormonal abnormalities that promote tumor proliferation [1-8,16-21]. Other hypotheses include the overproduction of reactive oxygen species during fatty acid metabolism, which might overwhelm the body's defensive mechanisms and set off the autoimmune response [15-22]. These excess oxidative stresses may activate many transcription factors, including those that control the expression of genes implicated in pro-inflammatory pathways. The effect of polyunsaturated fatty acids (PUFAs) on cancer risk has been shown to depend on the ratio of -6 to -3 PUFAs. In vivo findings demonstrated that -6 PUFAs stimulate tumor development, while -3 PUFAs are protective.

The statistically significant findings in this meta-analysis linking a high-fat diet to breast cancer can be compared to numerous other archival findings that echo a similar result. Even though these studies are not without confounders, they provided useful insight into this burning topic. First, some pre-clinical studies documented the positive association between a high-fat diet and breast cancer carcinogenesis [23-27]. For example, Cowen et al., in their attempt to explain the molecular basis for this phenomenon, designed a high-fat diet (60% kcal of fat) vs. a low-fat diet (16% kcal of fat) [23]. They observed that primary mammary fat and tumor growth rates were higher in the HF-fed diet group compared to the control group. This increased tumor rate was also associated with increased adipose tissue deposition, increased body weight gain, and increased proinflammatory cytokines (pronounced macrophage infiltration), leptins, and other pro-angiogenic factors (monocyte chemoattractant protein 1) [23-27]. These studies provided preclinical evidence suggesting that the HF diet induces mammary adipose tissue deposition and that obesity provides a pro-inflammation response that creates a favorable microenvironment for breast cancer carcinogenesis [23-27].

Human studies have demonstrated an association between a high-fat diet and breast cancer incidence. Nonetheless, the outcomes of human trials were not as substantial as those of preclinical investigations [1-8,16-21,24,28-31]. Ultimately, the discussion and study of the definitive nature of these complex interactions continue. Several studies have shown a link between a high-fat diet and obesity, and subsequently, obesity and breast cancer incidence, particularly in the postmenopausal population [7-16]. For example, Sauter and his colleagues [24] demonstrated that proteins linked to breast cancer are linked to BMI. In a pooled analysis of breast cancer patients and at-risk groups, they looked at several of these BMI-breast cancer-associated proteins [24]. First, prostate-specific antigen (PSA), a well-known tumor marker produced not just only in the prostate but also in normal and malignant breast tissue, was one of the tumor markers studied using multivariate classification and regression tree statistical models [24]. They documented that the levels of PSA in nipple aspirate fluid directly correlated to the incidence of breast cancer in these about 92% of the sample studied [24]. Other serum biomarkers studied in their breast cancer prevention studies were urinary plasminogen activator and basic fibroblast growth factor, all of which had similar positive correlations with breast cancer. They also noted that women with high BMIs under 45 are likelier to have ER-aggressive tumors than their thinner age-matched cohorts and that women who gain weight after menopause are more likely to develop estrogen/progesterone (ER/PR)-positive breast cancer than ER/PR-negative breast cancer [24]. Secondly, in their effort to explain the intricate relationships between obesity and breast cancer incidence, the National Cancer Institute documented that obese or overweight individuals had postmenopausal breast cancer rates that are 1.2-1.4 times higher than the general population, increasing by 1.2 times for every five-unit rise in BMI. While in the premenopausal group, those who are overweight or obese had 0.8 times higher risk of developing breast cancer [8-31]. Some adipokines, such as leptin, and other cell growth regulators, such as AMP-activated protein kinase and mammalian target of rapamycin (mTOR), show positive correlations with increasing body fat and may encourage abnormal cell proliferation, which helps explain the pathophysiology [31]. Furthermore, a 2014 study with a sample size of about 300,000 across Europe designed to look at several relations between lifestyle and cancer, including diet, published an association between HER2-negative breast cancer and hormone receptor-positive breast cancer, particularly saturated fats [22].

Limitations of this study

According to the National Cancer Institute, a limited number of these studies show the strength of the association [31], and many of them may have biases associated with observational studies. Furthermore, the majority of these observational studies and the data from these types of studies cannot explain this association categorically, as it is possible that people who are overweight, underweight, obese, or within normal weight ranges may differ in other ways that explain their increased risk for breast cancer other than their body fat.

## Conclusions

Obesity is a risk factor for breast cancer. Several theories have been given to explain this relationship, including the role of adipose tissues in producing estrogen, which plays a role in tumorigenesis. High-fat diets can result in obesity. However, the relationship between a high-fat diet as a risk factor for breast cancer is still complex. Not all existing studies demonstrate a strong association. This meta-analysis sought to determine the strength of the association between a high-fat diet and breast cancer risk while reviewing existing observational studies. The type of fat consumed has been found to determine to what extent the risk of breast cancer is increased. Hence, this paper also looked at various kinds of lipids and fats consumed in diets, including oils and polyunsaturated, saturated, trans, and monounsaturated fats, and their association with breast cancer risk. Though the studies were observational, with possible confounders, we sequenced data for the meta-analysis and determined some statistically significant positive correlations between a "high-fat" diet and the incidence of breast cancer. A healthy diet indeed includes eating foods that are rich in fats. However, the effect of consuming "excess fat" as a probable cause of breast cancer continues to accrue statistical strength. While other stronger risk factors for breast cancer exist, including age, sex, and genetics, dietary counseling must not be neglected, as making nutritional modifications that favor a healthy lifestyle can significantly reduce breast cancer risk and, thus, mortality and morbidity from the disease. Historically, scientific arguments and empirical data have suggested that high consumption of "bad fats," such as saturated and trans fats, may have a direct association with the development of breast cancer; however, quantitative data are still lacking in the discussion. Because of the scarcity of data from well-designed randomized controlled interventional trials to support or refute the findings of these observational studies, there is room for more research in this area of interest.

## References

[1] (2022). World Cancer Research Fund International. Breast cancer statistics. http://www.wcrf.org/dietandcancer/cancer-trends/breast-cancer-statistics.

[2] (2022). CDC. Basic information about breast cancer. https://www.cdc.gov/cancer/breast/basic_info/index.htm.

[3] Ziegler RG, Hoover RN, Pike MC (1993). Migration patterns and breast cancer risk in Asian-American women. J Natl Cancer Inst.

[4] Thiébaut AC, Rosenberg PS, Thompson FE, Hollenbeck AR (2006). Dietary fat intake and breast cancer risk in the NIH-AARP diet and health study. Am J Epidemiol.

[5] Murtaugh MA, Sweeney C, Giuliano AR (2008). Diet patterns and breast cancer risk in Hispanic and non-Hispanic white women: the Four-Corners Breast Cancer Study. Am J Clin Nutr.

[6] Bray GA, Popkin BM (1998). Dietary fat intake does affect obesity!. Am J Clin Nutr.

[7] Costa I, Moral R, Solanas M, Andreu FJ, Ruiz de Villa MC, Escrich E (2011). High corn oil and extra virgin olive oil diets and experimental mammary carcinogenesis: clinicopathological and immunohistochemical p21Ha-Ras expression study. Virchows Arch.

[8] Fung TT, Hu FB, Hankinson SE, Willett WC, Holmes MD (2011). Low-carbohydrate diets, dietary approaches to stop hypertension-style diets, and the risk of postmenopausal breast cancer. Am J Epidemiol.

[9] Kruk J, Marchlewicz M (2013). Dietary fat and physical activity in relation to breast cancer among Polish women. Asian Pac J Cancer Prev.

[10] Jones JA, Hartman TJ, Klifa CS (2015). Dietary energy density is positively associated with breast density among young women. J Acad Nutr Diet.

[11] Berkey CS, Tamimi RM, Willett WC (2019). Dietary intake from birth through adolescence in relation to risk of benign breast disease in young women. Breast Cancer Res Treat.

[12] Dydjow-Bendek D, Zagoźdźon P (2020). Total dietary fats, fatty acids, and Omega-3/Omega-6 ratio as risk factors of breast cancer in the Polish population: a case-control study. In Vivo.

[13] Khankari NK, Bradshaw PT, Steck SE (2015). Polyunsaturated fatty acid interactions and breast cancer incidence: a population-based case-control study on Long Island, New York. Ann Epidemiol.

[14] Link LB, Canchola AJ, Bernstein L, Clarke CA, Stram DO, Ursin G, Horn-Ross PL (2013). Dietary patterns and breast cancer risk in the California Teachers Study cohort. Am J Clin Nutr.

[15] Turati F, Carioli G, Bravi F (2018). Mediterranean diet and breast cancer risk. Nutrients.

[16] Bojková B, Winklewski PJ, Wszedybyl-Winklewska M (2020). Dietary fat and cancer—which is good, which is bad, and the body of evidence. Int J Mol Sci.

[17] Field CJ, Robinson L (2019). Dietary fats. Adv Nutr.

[18] (2022). Diet high in saturated fat linked to higher risk of HER2-negative, hormone-receptor-positive breast cancer. https://www.breastcancer.org/research-news/high-fat-diet-linked-to-breast-cancer.

[19] Matta M, Huybrechts I, Biessy C (2021). Dietary intake of trans fatty acids and breast cancer risk in 9 European countries. BMC Med.

[20] Bassett JK, Hodge AM, English DR, MacInnis RJ, Giles GG (2016). Plasma phospholipids fatty acids, dietary fatty acids, and breast cancer risk. Cancer Causes Control.

[21] Ozobokeme OE, Jones TE, Naous R, Khader SN (2022). Educational Case: Ewing sarcoma family of tumors: clinical presentation, pathologic findings, and differential diagnosis. Acad Pathol.

[22] Evbayekha EO, Okobi OE, Okobi T (2022). The evolution of hypertension guidelines over the last 20+ years: a comprehensive review. Cureus.

[23] Cowen S, McLaughlin SL, Hobbs G, Coad J, Martin KH, Olfert IM, Vona-Davis L (2015). High-fat, high-calorie diet enhances mammary carcinogenesis and local inflammation in MMTV-PyMT mouse model of breast cancer. Cancers (Basel).

[24] Sauter ER, Scott S, Hewett J, Kliethermes B, Ruhlen RL, Basarakodu K, de la Torre R (2008). Biomarkers associated with breast cancer are associated with obesity. Cancer Detect Prev.

[25] Vona-Davis L, Rose DP (2013). The obesity-inflammation-eicosanoid axis in breast cancer. J Mammary Gland Biol Neoplasia.

[26] Yoshimura T, Howard OM, Ito T (2013). Monocyte chemoattractant protein-1/CCL2 produced by stromal cells promotes lung metastasis of 4T1 murine breast cancer cells. PLoS One.

[27] Wagner M, Bjerkvig R, Wiig H, Melero-Martin JM, Lin RZ, Klagsbrun M, Dudley AC (2012). Inflamed tumor-associated adipose tissue is a depot for macrophages that stimulate tumor growth and angiogenesis. Angiogenesis.

[28] Gopinath A, Cheema AH, Chaludiya K (2022). The impact of dietary fat on breast cancer incidence and survival: a systematic review. Cureus.

[29] Sieri S, Chiodini P, Agnoli C (2014). Dietary fat intake and development of specific breast cancer subtypes. J Natl Cancer Inst.

[30] Schulz M, Hoffmann K, Weikert C, Nöthlings U, Schulze MB, Boeing H (2008). Identification of a dietary pattern characterized by high-fat food choices associated with increased risk of breast cancer: the European Prospective Investigation into Cancer and Nutrition (EPIC)-Potsdam Study. Br J Nutr.

[31] (2022). National Cancer Institute. Obesity and cancer. https://www.cancer.gov/about-cancer/causes-prevention/risk/obesity/obesity-fact-sheet.

